# Association between telomere length and hepatic fibrosis in non-alcoholic fatty liver disease

**DOI:** 10.1038/s41598-021-97385-2

**Published:** 2021-09-09

**Authors:** Hee Kyung Shin, Jeong Hwan Park, Jung Hwan Yu, Young-Joo Jin, Young Ju Suh, Jin-Woo Lee, Won Kim, The Korean Nonalcoholic Fatty Liver Study Group (KNSG)

**Affiliations:** 1 Department of Internal Medicine, Inha University Hospital, Inha University School Medicine, Inchon, South Korea; 2 Department of Pathology, Seoul National University College of Medicine, Seoul Metropolitan Government Boramae Medical Center, Seoul, Korea; 3 Department of Biomedical Sciences, College of Medicine, Inha University, Inchon, South Korea; 4 Division of Gastroenterology and Hepatology, Department of Internal Medicine, Seoul National University College of Medicine, Seoul Metropolitan Government Boramae Medical Center, Seoul, Korea

**Keywords:** Hepatology, Endocrine system and metabolic diseases, Gastrointestinal diseases

## Abstract

Telomere length has been linked to the prevalence and progression of metabolic disease. However, clinical implications of telomere length in biopsy-proven non-alcoholic fatty liver disease (NAFLD) patients remain unclear. Therefore, this study aimed to investigate the association of telomere length with the histological severity of NAFLD. The cross-sectional data derived from the prospectively enrolled Boramae NAFLD registry (n = 91) were analyzed. The liver tissues and clinical information were obtained from both NAFLD patients and non-NAFLD subjects. Binary logistic regression was performed to identify the independent association between telomere length and the histological severity of NAFLD. A total of 83 subjects with or without biopsy-proven NAFLD were included for analysis: non-NAFLD in 23 (27.7%), non-alcoholic fatty liver in 15 (18.1%), and non-alcoholic steatohepatitis (NASH) in 45 (54.2%). Telomere length measured from liver tissues showed a strong negative correlation (*p* < 0.001) with age, regardless of NAFLD status. Therefore, telomere length was corrected for age. Age-adjusted telomere length than decreased gradually with an increasing severity of fibrosis in patients with NAFLD (*p* < 0.028). In multivariate analysis, age-adjusted telomere length (odds ratio [OR] 0.59; 95% CI 0.37–0.92; *p* = 0.019) and high-density lipoprotein cholesterol (OR 0.94; 95% CI 0.80–0.99; *p* = 0.039) were independently associated with significant fibrosis. The age-adjusted telomere length tends to decrease along with the fibrosis stage of NAFLD. In particular, among the histological components of NAFLD, fibrosis severity seems to be related to telomere length in the liver.

## Introduction

The telomere is a region of repetitive nucleotide sequences at each end of a chromosome, which protects the chromosome tips from end-to-end fusion, translocation, and rearrangement^1^. Telomere length decreases gradually during mitosis. Hence, the shortening of telomere length is accepted as a process of somatic cells aging^1^. Thus, telomere shortening is a phenomenon involved in cell survival and death. On the other hand, in some immortal cells, such as cancer cell, the telomere's original function is restricted because telomere length is repaired. Furthermore, the dysfunction and mutation of telomerase involved in telomere length are closely related to various chronic diseases, including non-alcoholic fatty liver disease (NAFLD)^1^.

NAFLD is the most common chronic liver disease, affecting approximately a quarter of the global adult population^2^. Non-alcoholic fatty liver (NAFL) can progress to liver cirrhosis and hepatocellular carcinoma (HCC) via multiple metabolic and molecular pathways^3^. Moreover, NAFLD poses major public health problems causing an increasing economic burden^4^. Therefore, many studies have been conducted to prevent serious complications, such as decompensated cirrhosis and HCC, by understanding molecular mechanisms underlying the progression of NAFLD^5^. Since NAFLD involves multiple environmental and genetic factors, various approaches and studies to understand the pathophysiology of NAFLD progression are needed (e.g., telomere shortening).

According to a previous study of the relationship between telomere length and NAFLD, telomere length measured in leukocytes isolated from the peripheral blood of NAFLD patients was an independent risk factor for advanced fibrosis^1,6,7^. However, that study evaluated advanced fibrosis using hematologic biomarkers, such as the NAFLD fibrosis score and Fibrosis-4 (FIB-4) index. In the liver tissues of NAFLD patients, shorter telomere length and increased cellular senescence were observed, but most studies examined a small number of samples, and careful analysis of the pathology was not performed. Although several studies have been conducted to reveal the association between NAFLD and telomere length, the relationship remains elusive due to the various limitations of each study.

Therefore, the current study examined the relationship between NAFLD and telomere length using liver tissues obtained from NAFLD patients and non-NAFLD subjects. The aim of this study was to identify the clinical relevance of the change in telomere length according to the histological severity of NAFLD.

## Material and methods

### Study subjects

**Figure 1:**
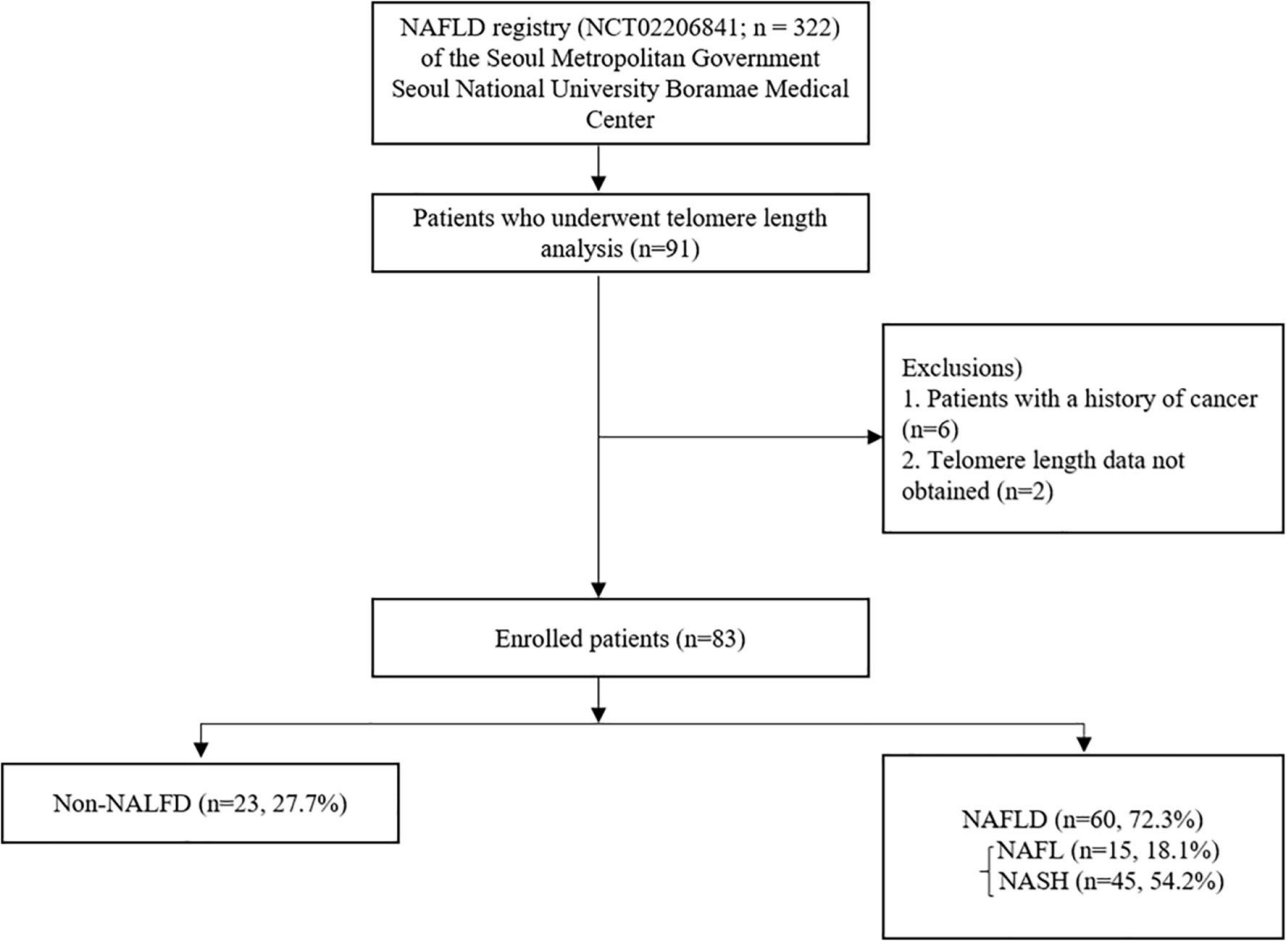
Study flow diagram. *NAFLD* non-alcoholic fatty liver disease, *NAFL* non-alcoholic fatty liver, *NASH* non-alcoholic steatohepatitis.

We used a prospectively enrolled cross-sectional cohort derived from the ongoing NAFLD registry (NCT02206841; n = 322) of the Seoul Metropolitan Government Seoul National University Boramae Medical Center from January 2013 to January 2017. The inclusion criteria were adults (age ≥ 18 years) and patients suspected of NAFLD who underwent liver biopsy and confirmed by pathologic evaluation. Control liver tissues were obtained from subjects who underwent a liver biopsy to characterize a solid liver mass suspected of hepatic adenoma or focal nodular hyperplasia, based on radiographic findings without any evidence of hepatic steatosis. In addition, control liver tissues were also collected through a pre-evaluation of liver transplant donors. The DNA required for telomere analysis was extracted from the liver tissues of 91 subjects, and their telomere length was measured. Among them, two subjects with no telomere length data, and six subjects with a previous history of cancer were excluded from analysis. Finally, 83 subjects were included in the current study (Fig. 1). The subjects were examined for height, weight, body mass index (BMI), drug and alcohol history, and other medical history through history taking and a physical examination. The complete blood counts, liver function tests, and biochemistry analyses (total cholesterol, triglycerides, high-density lipoprotein cholesterol, low-density lipoprotein cholesterol, total protein, albumin, fasting glucose, and fasting insulin, etc.) were performed. Lipoprotein profile tests were also conducted. Liver stiffness and controlled attenuation parameter (CAP) were measured by two experienced operators using vibration-controlled transient elastography (FibroScan; Echosens, Paris, France) according to the manufacturer’s recommendations. Vibration-controlled transient elastography was performed using the M or XL probe after fasting for at least 4 h within one month of percutaneous liver biopsy. As described elsewhere, unreliable data were defined as an interquartile range (IQR) per median of LSM (IQR/M) > 0.3 with a median LSM ≥ 7.1 kPa, and those unreliable results were excluded from analysis^8^. Informed consent was obtained from all participants under a protocol approved by the institutional review board (#20130320/16-2013-45/041). The study was conducted in accordance with the Declaration of Helsinki and approved by the Institutional Review Board of Inha University Hospital, Incheon, South Korea (Approval number: INHAUH 2018-06-033-006).

### Telomere length analysis

Telomere length was measured with an Absolute Human Telomere Length Quantification qPCR Kit (#8918) by ScienCell using relative quantification, one of the qPCR quantification methods. Telomere length measurement was performed by confirming the Ct value using a primer set for quantifying the telomere sequence section per sample, including the reference cell line DNA, and confirming the Ct value using a primer set for quantifying a single cell copy. The experiment was conducted on a 20 μL sample, including 2 × qPCR master mix 10 μL per reaction, Primer stock solution (Telomere or Single cell primer stock) 2 μL, and Genetic DNA template 0.5–5 ng. The qPCR protocol was carried out in 35 cycles after 10 min of initial denaturation at 95 °C, and 35 cycles of denaturation at 95 °C for 20 s, annealing at 52 °C for 20 s, and extension at 72 °C for 45 s. In this process, we did not differentiate between non-parenchymal and parenchymal cells in the liver using microdissection when we had obtained telomere length values. The hepatocytes that display telomere shortening in the liver constitute 80% of the entire liver volume. Thus, the results of this study may be attributed mainly to the telomere length in the hepatocytes.

### Liver histology

Liver specimens were obtained using 16 G disposable needles, fixed in 4% formalin, and embedded in paraffin. Adequate specimens needed to be at least 20 mm in length. The sections (3 mm thick) were stained with hematoxylin–eosin and Masson’s trichrome. The control liver tissues were collected from subjects who underwent a liver biopsy to characterize a solid liver mass suspected of hepatic adenoma or focal nodular hyperplasia, based on the radiographic findings without any evidence of hepatic steatosis. In addition, control liver tissues were also collected through a pre-evaluation of liver transplant donors. A single experienced liver pathologist assessed and reviewed all liver biopsies. The pathologist was blind in all clinical data including telomere length. NAFLD was defined as the presence of ≥ 5% macrovesicular steatosis^9^. Non-alcoholic steatohepatitis (NASH) was diagnosed based on the overall pattern of histological hepatic injury consisting of macrovesicular steatosis, inflammation, and hepatocellular ballooning according to Brunt et al.’s criteria^10,11^. Therefore, although there were some cases with nonalcoholic fatty liver (i.e., simple steatosis) that were not accompanied by ballooning or inflammation, NASH was defined as ballooning of score 1 or greater. All of these NAFLD classifications have been made according to the NASH CRN histological scoring system. Our study classified the degree of fibrosis based on Masson's trichrome staining, and fibrosis severity was assessed using a five-point scale proposed by Brunt and modified by Kleiner et al. and cirrhosis was subdivided into F4A, F4B, and F4C according to the Laennec classification: F0, absence of fibrosis; F1a-c, perisinusoidal or periportal fibrosis; F2, perisinusoidal and portal/periportal fibrosis; F3, bridging fibrosis; and F4A-C, cirrhosis^12,13^. Significant fibrosis was defined as F2–F4 (Supplementary Fig. S1). Also, immunohistochemical study to assess proliferation of hepatocytes and degree of inflammation was performed using proliferating cell nuclear antigen (PCNA) and CD68 antibodies on selected cases.

### Immunohistochemical study

Selected liver biopsy samples from both longer telomere (n = 3) and shorter telomere (n = 3) cases were performed immunohistochemical analysis. The proliferating cell nuclear antigen (PCNA) and CD68 antibodies were used to assess proliferation of hepatocytes and degree of inflammation, respectively. Immunostaining of PCNA was performed using BenchMark ULTRA (Ventana Medical System, Inc., Tucson, AZ) and polyclonal rabbit anti-PCNA antibody (Abcam, Cambridge, UK) with dilution of 1:20,000. The Optiview universal DAB kit (Ventana Medical Systems, Inc., Tucson, AZ) was used to detect primary antibody, according to manufacturer’s protocol. The nuclear staining was regarded as positive staining. In case of CD 68 immunostaining, Dako Omnis (Dako, Glostrup, Denmark) and monoclonal mouse anti-CD68 antibody (Leica Biosystems, IL) with dilution of 1:50 were used. The EnVision FLEX (Dako, Glostrup, Denmark) was used as a detection kit, according to manufacturer’s instruction. The cytoplasmic staining was considered positive staining and Kupffer cells were indicated as a normal positive control.

### Statistical analysis

Baseline clinical characteristics are described as mean ± standard deviation, and categorical variables are expressed as percentages. Univariable comparisons between the two groups were made using Student’s t test for continuous variables and the χ^2^ test for categorical variables as appropriate. In this study, the age-adjusted telomere length was calculated by considering the age affecting telomere length. To determine the age-adjusted telomere length, telomere was regressed according to age, and residual telomere was obtained. Using the regression analysis on telomere of age, the values of the telomere adjusted for age were estimated as follows: age-adjusted telomere length = telomere − 7.668 + 0.043 × age. The association between telomere length and significant fibrosis was assessed by multivariable binary logistic regression to adjust for clinical and laboratory confounding factors. Significance was defined as *p* < 0.05. Statistical analysis was performed using SPSS v19.0 (SPSS Inc., Chicago, IL, USA).

## Results

### Baseline characteristics

Baseline characteristics of study subjects are presented in Table 1. The median age of study subjects was 54 years (19–80), and 41 subjects (48.8%) were male. The median BMI (kg/m^2^) was 26.8 (19.1–39.1), liver stiffness (kPa) was 7.62 (3.2–35.8), controlled attenuation parameter (dB/m) was 285.1 (144–398), and homeostasis model assessment of insulin resistance (HOMA-IR) was 5.47 (0–22.02). Among the 83 subjects, 23 (27.7%) were classified as non-NALFD, and 60 (72.3%) had NAFLD (NAFL, n = 15, 18.1%; NASH, n = 45, 54.2%) (Fig. 1). The prevalence of gender and type 2 diabetes mellitus (DM), BMI, liver stiffness, controlled attenuation parameter, alanine aminotransferase (ALT), aspartate aminotransferase (AST), gamma-glutamyl transferase (γGT), glucose, triglycerides, and HOMA-IR were significantly different between the two groups.

**Table 1:** Clinical and laboratory characteristics of study subjects.

	Total (n = 83)	Non-NAFLD (n = 23)	NAFLD (n = 60)	*P* value
Age, years (SD)	54.5 (± 14.3)	57.5 (± 11.1)	53.3 (± 15.3)	0.176
Male, n (%)	41 (48.8)	15 (65.2)	26 (43.3)	0.076
Hypertension, n (%)	37 (44.0)	8 (34.8)	29 (48.3)	0.268
DM, n (%)	33 (39.2)	5 (21.7)	28 (46.7)	0.027
BMI (kg/m^2^)	26.8 (± 4.4)	23.6 (± 2.9)	28.0 (± 4.3)	< 0.001
LS (kPa)	7.62 (± 5.7)	4.82 (± 1.2)	8.55 (± 6.3)	< 0.001
CAP (dB/m)	285.1 (± 60.9)	214.5 (± 43.7)	308.6 (± 45.9)	< 0.001
WBC (/μL)	6143 (± 1834)	5753 (± 1717)	6290 (± 1869)	0.233
Hb (g/dL)	13.5 (± 2.1)	12.8 (± 2.4)	13.7 (± 1.9)	0.086
Platelet (× 10^3^**/**μL)	222 (± 70.9)	222 (± 62.6)	222 (± 74.4)	0.988
Total bilirubin (mg/dL)	0.8 (± 0.3)	0.8 (± 0.3)	0.8 (± 0.4)	0.430
AST (IU/L)	56.5 (± 56.0)	26.5 (± 8.9)	67.9 (± 62.1)	< 0.001
ALT (IU/L)	70.7 (± 84.2)	24.5 (± 10.5)	88.5 (± 93.1)	< 0.001
ALP (IU/L)	84.8 (± 30.0)	77.1 (± 24.5)	87.7 (± 31.5)	0.152
GGT (IU/L)	59.6 (± 43.1)	40.2 (± 41.9)	67.2 (± 41.6)	0.010
Creatinine (mg/dL)	0.77 (± 0.2)	0.82 (± 0.2)	0.75 (± 0.2)	0.218
Glucose (mg/dL)	127.6 (± 48.9)	112.0 (± 27.5)	133.6 (± 54.0)	0.020
Cholesterol (mg/dL)	174.9 (± 46.0)	163.9 (± 45.4)	179.2 (± 46.0)	0.181
TG (mg/dL)	146.6 (± 77.7)	112.2 (± 45.4)	160.0 (± 83.7)	0.011
HDL (mg/dL)	45.3 (± 12.8)	44.7 (± 12.1)	45.5 (± 13.1)	0.790
LDL (mg/dL)	98.1 (± 34.6)	94.6 (± 35.5)	99.6 (± 34.5)	0.620
HOMA-IR	5.47 (± 4.5)	2.85 (± 1.1)	6.48 (± 4.9)	< 0.001

Data are expressed as mean (SD).

*DM* diabetes mellitus, *BMI* body mass Index, *LS* liver stiffness, *CAP* controlled attenuation parameter, *WBC* white blood cell, *Hb* hemoglobin, *AST* aspartate aminotransferase, *ALT* alanine aminotransferase, *ALP* alkaline phosphatase, *GGT* gamma-glutamyl transferase, *TG* triglycerides, *HDL* high-density lipoprotein, *LDL* low-density lipoprotein, *NAFLD* non-alcoholic fatty liver disease, *HOMA-IR* homeostasis model assessment of insulin resistance.

### Telomere length and fibrosis severity of NAFLD

**Figure 2:**
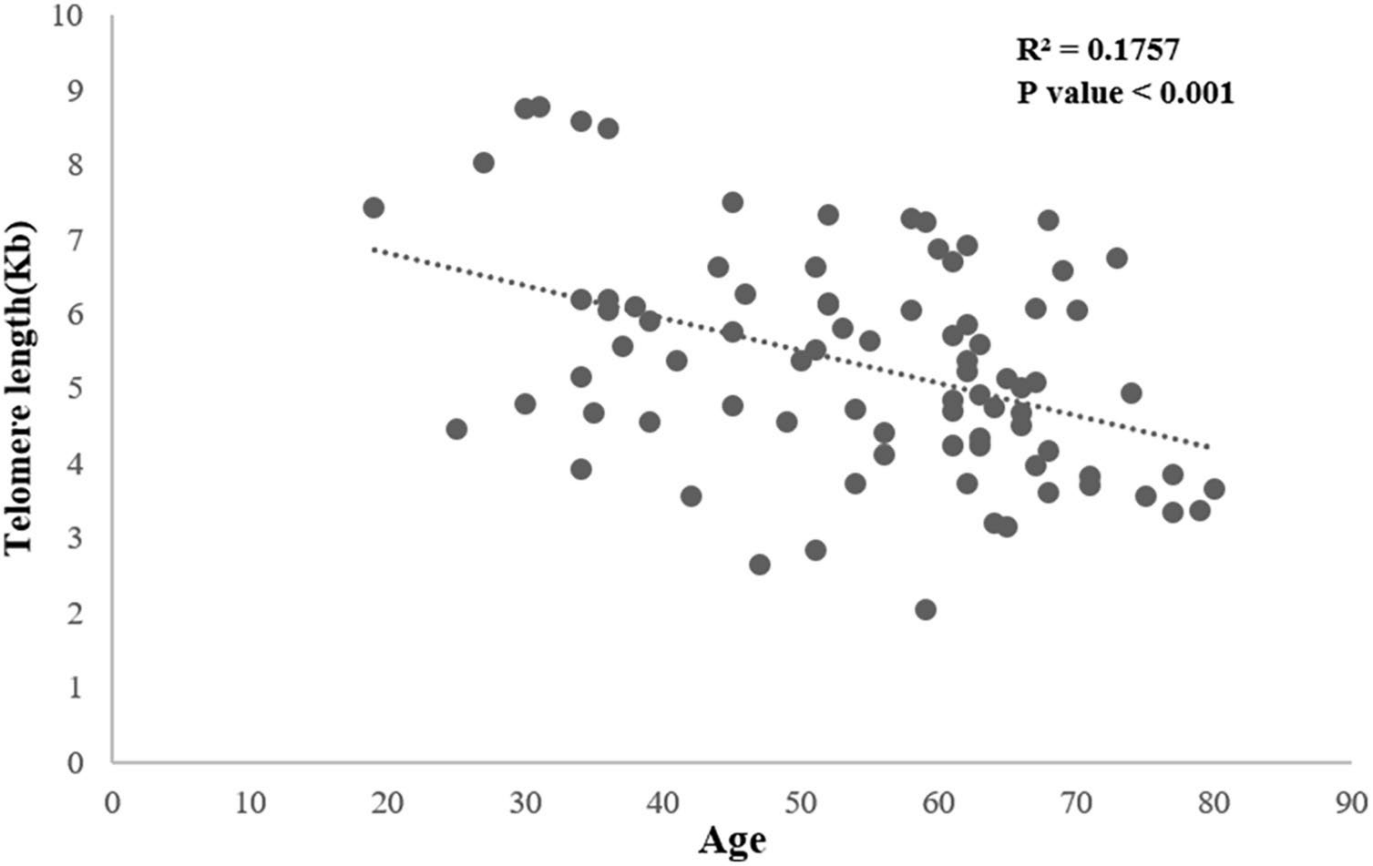
Correlation between telomere length and age. There was a strong negative correlation (*p* < 0.001) between telomere length and age.

The median telomere length was 5.15 Kb (2.03–8.77). A strong negative correlation (*p* < 0.001) between telomere length and age was observed, regardless of NAFLD status (Fig. 2). Therefore, the age-adjusted telomere length was calculated using linear regression analysis with age and telomere as the independent variables. The age-adjusted telomere length was calculated to determine its relationship with fibrosis stage, steatosis grade, lobular inflammation score, and ballooning score. The age-adjusted telomere length ranged from − 3.1 to 2.5 with a median of − 0.08.

**Figure 3:**
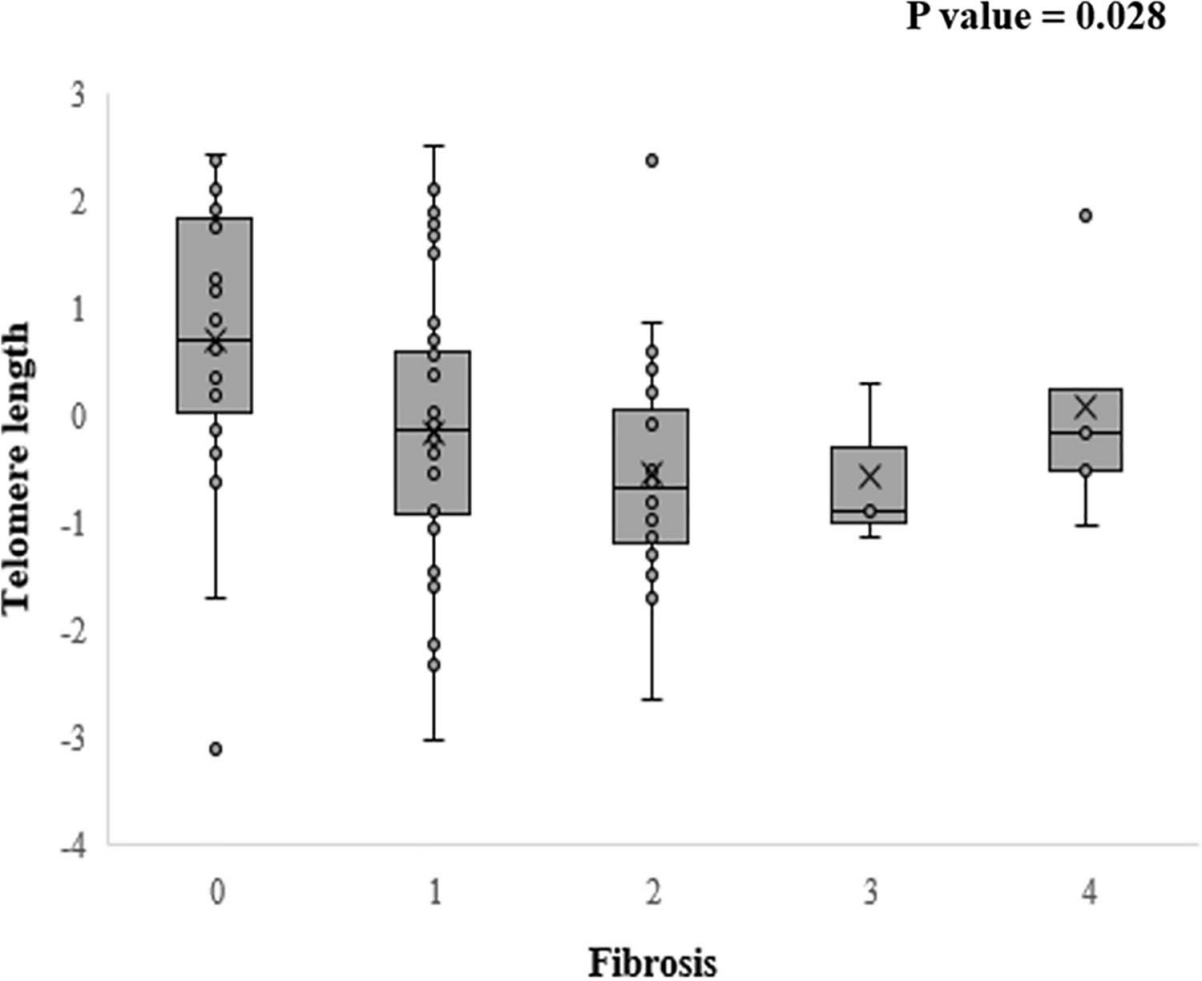
Correlation between telomere length and the ALT level. (**A**) Correlation between telomere length and ALT level: telomere length was shorter in the elevated ALT group than in the normal ALT group (elevated ALT; male > 34 IU/L, female > 26 IU/L). (**B**) Correlation between the telomere length and ALT values (continuous variable). ALT values and telomere length showed a marginal inverse correlation, which was not statistically significant.; *ALT* alanine aminotransferase.

This study examined the relationship between telomere length and the histological components of NAFLD, such as fibrosis, steatosis, lobular inflammation, and ballooning. Telomere length significantly decreased with increasing fibrosis severity in NAFLD patients (*p* = 0.028) (Fig. 3). When fibrosis was assessed based on a five-point scale from F0 to F4, the median telomere lengths of the F0, F1, F2, F3 and F4 groups were 0.70, − 0.13, − 0.68, − 0.88 and − 0.55, respectively. However, there was no significant correlation between telomere length and the other histological components of NAFLD, such as steatosis, lobular inflammation, and ballooning (Supplementary Fig. S2). When dividing study subjects into two groups with telomere length shorter or longer than the average value (− 0.013), the stage of fibrosis was significantly higher in the group with shorter telomere length (*p* = 0.043). Nevertheless, the other histological components, such as steatosis, lobular inflammation, and ballooning, showed no significant difference between the two groups (Supplementary Table S1).

### Correlation between telomere length and ALT levels in NAFLD patient

**Figure 4:**
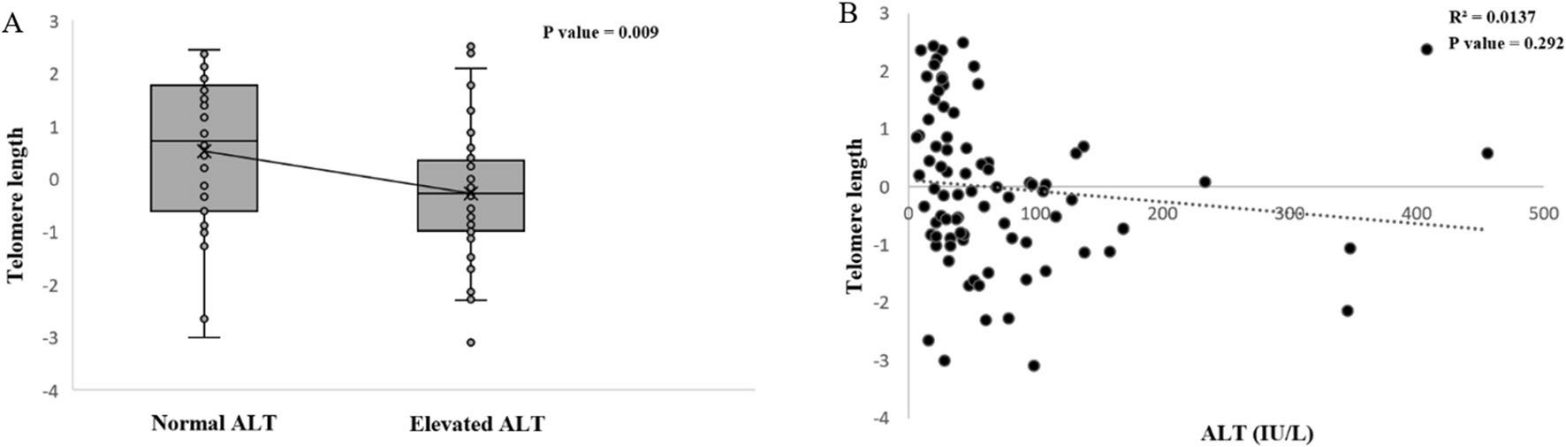
Correlation between telomere length and fibrosis stage. Telomere length was decreased along with increasing fibrosis severity in NAFLD patients (*p* = 0.028); *NAFLD* non-alcoholic fatty liver disease.

This study investigated the relationship between telomere length and liver injury using ALT levels, as the inflammation generated by chronic liver injury may constitute the actual trigger of telomere shortening. First, we investigated the correlation between ALT and telomere length by dividing study participants into groups of normal ALT and elevated ALT (elevated ALT, male > 34 IU/L, female > 26 IU/L). Telomere length was significantly shorter in the elevated ALT group than in the normal ALT group (*p* = 0.009) (Fig. 4a). Furthermore, the correlation between telomere length and ALT value (continuous variable) was investigated, and the ALT value and telomere length showed marginal inverse correlation, but were not statistically significant (*p* = 0.292) (Fig. 4b).

### Independent predictors factor of significant fibrosis in NAFLD patient

In the logistic regression analysis to find factors associated with significant fibrosis (≥ F2) in NAFLD patients, the alkaline phosphatase (ALP), glucose, high-density lipoprotein (HDL) cholesterol, hypertension, type 2 diabetes mellitus and age-adjusted telomere length were significantly associated with significant fibrosis (*p* ≤ 0.05, respectively). In multivariable analysis based on factors that were meaningful in univariable analysis, the age-adjusted telomere length (odds ratio [OR] 0.59; 95% CI 0.37–0.92; *p* = 0.019) and high-density lipoprotein cholesterol (OR 0.94; 95% CI 0.80–0.99; *p* = 0.039) were independently associated with significant fibrosis in NAFLD patients (Table 2).

**Table 2:** Independent predictors of significant fibrosis in NAFLD patients.

Variables	Univariable analysis		Multivariable analysis	
	OR (95% CI)	*P* value	OR (95% CI)	*P* value
Male, n (%)	0.54 (0.21–1.36)	0.191		
Hypertension, n (%)	2.71 (1.06–6.90)	0.037		
DM, n (%)	2.98 (1.16–7.65)	0.023		
Alcohol, n (%)	0.41 (0.12–1.36)	0.144		
BMI (kg/m^2^)	1.10 (0.99–1.22)	0.073		
WBC (/μL)	0.96 (0.75–1.23)	0.744		
Hb (g/dL)	0.96 (0.77–1.19)	0.704		
Platelet (× 10^3^/μL)	0.99 (0.99–1.00)	0.066		
Total bilirubin (mg/dL)	1.445 (0.39–5.41)	0.584		
AST (IU/L)	1.00 (1.00–1.01)	0.277		
ALT (IU/L)	1.00 (1.00–1.01)	0.536		
ALP (IU/L)	1.02 (1.00–1.04)	0.015		
GGT (IU/L)	1.01 (1.00–1.02)	0.053		
Creatinine (mg/dL)	0.24 (0.02–2.64)	0.241		
Glucose (mg/dL)	1.01 (1.00–1.02)	0.038		
TG (mg/dL)	1.00 (1.00–1.01)	0.514		
Cholesterol (mg/dL)	0.99 (0.98–1.00)	0.216		
HDL (mg/dL)	0.94 (0.90–0.99)	0.012	0.94 (0.89–0.99)	0.039
LDL (mg/dL)	0.99 (0.97–1.00)	0.137		
Telomere length^a^	0.69 (0.48–1.00)	0.050	0.59 (0.37–0.92)	0.019

Table 2 enrolled parameters with *p* value ≤ 0.05 in univariate analysis into multivariate analysis.

a = age-adjusted telomere length.

*DM* diabetes mellitus, *BMI* body mass index, *WBC* white blood cell, *Hb* hemoglobin, *AST* aspartate aminotransferase, *ALT* alanine aminotransferase, *ALP* alkaline phosphatase, *GGT* gamma-glutamyl transferase, *TG* triglycerides, *HDL* high-density lipoprotein, *LDL* low-density lipoprotein.

### Telomere length across different histological spectra of NAFLD

**Figure 5:**
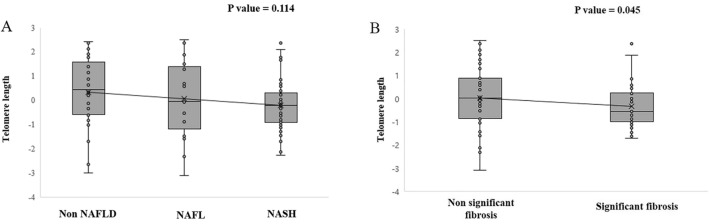
Correlation between telomere length and the histological spectrum of NAFLD. (**A**) Correlation between telomere length and NAFLD: telomere length tended to be shorter in the order of non-NAFLD, NAFL, and NASH (*p* = 0.114). (**B**) Correlation between telomere length and significant fibrosis: telomere length tended to be shorter in NAFLD patients with significant fibrosis than those without significant fibrosis (*p* = 0.045). *NAFLD* non-alcoholic fatty liver disease, *NAFL* non-alcoholic fatty liver, *NASH* non-alcoholic steatohepatitis.

When comparing telomere length according to the histological spectra of NAFLD, telomere length tended to be shorter in the order of non-NAFLD, NAFL, and NASH though it was not statistically significant (*p* = 0.114) (Fig. 5a). The median telomere lengths of the non-NAFLD, NAFL, and NASH groups were 0.44, − 0.04, and − 0.22, respectively. We then examined the difference in telomere length between subjects without and with significant fibrosis. The median telomere length was significantly shorter in NAFLD patients with significant fibrosis than those without significant fibrosis (− 0.57 vs. 0.03, *p* = 0.045) (Fig. 5b).

**Figure 6:**
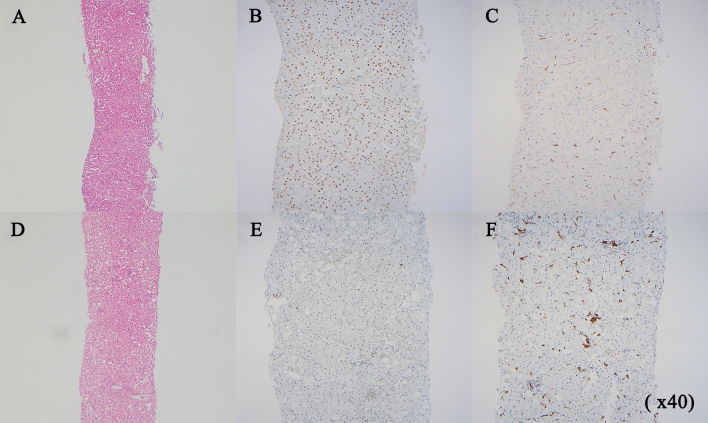
Representative photomicrograph and immunohistochemical results of NAFL and NASH patients. (**A**–**C**) NAFL patient with long telomere length [(**A**) H&E stain, (**B**) PCNA, (**C**) CD68)], (**D**–**F**) NASH patients with short telomere length [(**D**) H&E stain, (**E**) PCNA, (**F**) CD68)] [original magnification, (**A**,**D**), × 50; (**B**,**C**,**E**,**F**), × 100]; *NAFL* non-alcoholic fatty liver, *NASH* non-alcoholic steatohepatitis.

To determine the degree of proliferation and inflammation for each subgroup, we performed immunohistochemical staining using PCNA and CD68 antibodies. As a result, the NASH with short telomere length group showed a decrease in PCNA staining than the NAFL with long telomere length group, suggesting less proliferation. In CD68 staining used to confirm inflammation severity, the inflammatory cells were more abundant in the NASH with a short telomere length group than in the NAFL with a long telomere length group, which was in line with the histological findings (H&E). These results suggest that the decreased hepatocellular proliferation and regeneration along with telomere shortening are accompanied by persistent hepatocyte damage and inflammation as NAFL progresses to NASH and fibrosis (Fig. 6).

## Discussion

This study investigated the relationship between NAFLD and telomere length using the liver tissue samples obtained from study subjects with biopsy-proven NAFLD. Telomere length was shorter in NAFLD subjects than in non-NAFLD subjects. Moreover, telomere length decreased gradually with increasing severity of fibrosis in NAFL and NASH patients. In other studies, using liver tissues of NAFLD patients, shorter telomere length and increased cellular senescence were demonstrated in patients with NAFLD, but most of those studies had a small number of samples and the data were not comprehensively supported by extensive analysis of liver pathology^14–17^. Therefore, the relationship between the histological severity of NAFLD and telomere length needs to be further analyzed for clarification of the pathogenic role of telomere shortening in NAFLD progression. To this end, telomere length differences between the non-NAFLD and NAFLD (NAFL and NASH) groups were compared among the different spectra of NAFLD.

Telomeres are noncoding regions of repetitive nucleotide sequences (5′-TTAGGG-3′; telomeric repeats) located at the ends of chromosomes^18^. They participate in maintaining the integrity and stability of the genome during replication. Telomere length shortens each time a cell divides, and when a critical point is reached, the cell enters an inactive state such as cell cycle arrest or apoptosis^19^. Thus, telomere length is considered an indicator of the biological age of cells, and telomere shortening can be seen as a phenomenon occurring in all cellular tissues^18,19^. However, abnormalities in the normal telomere repair system and telomere shortening are often reported in chronic metabolic diseases, such as type 2 diabetes mellitus, cardiovascular disease, and NAFLD^20,21^. Moreover, previous reports have shown that changes in telomere length are of prognostic relevance^22^. Recently, many studies have been conducted to investigate the relationship between telomeres and chronic metabolic diseases^20^. In particular, there is considerable interest in the relationship between telomere length and NAFLD.

Previous studies on the relationship between telomeres and NAFLD revealed telomere dysfunction in patients with cryptogenic cirrhosis and NAFLD compared to non-NAFLD controls. Telomere length was shorter in the NAFLD group than in the healthy group^10^. On the other hand, most of these studies had several limitations by not applying proper age correction, which inversely correlates with telomere length. In the previous study, to compensate for these shortcomings, NAFLD patients were divided into three age categories (20–35 years old, 35–45 years old, and 45–60 years old) to analyze their telomere length. Telomere length by the age group decreased compared to normal subjects, but that study analyzed telomere length of peripheral blood leukocytes rather than liver tissues^23^. In contrast to previous studies, the current study confirmed the relationship between telomere length and the histological severity of NAFLD by adjusting telomere length for age and analyzed telomeres of hepatocytes rather than other resident cells.

Although the cause of telomere shortening in NAFLD remains unclear, oxidative stress and chronic inflammatory stress are thought to play an important role^6,23,24^. Oxidative stress causes double-strand breaks of the telomeric DNA, which consequently shortens its length. Furthermore, in chronic inflammatory conditions, the regenerative pressure associated with tissue regeneration increases, while telomere shortening is accelerated as it undergoes several cell divisions^22,25,26^. Consequently, chronic oxidative stress and inflammatory stress result in shortening of telomere length in NAFLD patients. Meanwhile, aging and high fat diet induce steatosis and senescence in normal hepatocytes through the p53-p21 and the p16-Rb pathway. These senescent cells secrete IL-1b, IL-6, chemokines, and ROS, leading both to reinforcement of telomere shortening in the neighboring cells and to disease progression to NASH. At the same time, senescent hepatocytes induce inflammation by secretion of interleukins and TNF, macrophage activation, and senescence of lymphocytes. Overall, these factors induce steatosis to normal hepatocytes, while favoring further progression of NAFL to NASH^27^.

Since telomere length is shortened in chronic diseases, such as NAFLD, telomere length may help predict the prognosis of chronic liver disease. Excessive telomere shortening due to a telomerase gene mutation or an acquired factor may impair the ability of hepatocytes to regenerate in response to chronic injury^28–30^, thereby promoting fibrosis progression and a shorter telomere length, resulting in worsening of fibrosis. A recent study on the relationship between telomere and HCC, one of the critical complications of chronic liver disease, confirmed that activated telomerase elongates the telomeres during HCC progression. Longer telomere lengths result in worse survival rates in HCC patients^31,32^. Therefore, telomere length be also used as a surrogate marker to predict the prognosis of patients with chronic liver disease, such as NAFLD.

The current study had some limitations. First, since it was a relatively small-scale study of only Asian ethnicity, the results of the current study might not be generalized to other ethnic populations. Therefore, further larger-scale studies are warranted to confirm our findings in the future. Second, sufficient DNA samples need to be secured for telomere analysis. Liver biopsy was performed for most patients with NAFLD, but it was difficult to obtain sufficient DNA samples. Therefore, it was not possible to measure telomere length ​​in all patients undergoing liver biopsy. Third, this study did not include magnetic resonance imaging proton density fat fraction (MR-PDFF) or MR spectroscopy to estimate fatty liver quantitatively^33^. Further studies using these tests would be helpful to examine the exact relationship between telomere length and quantitative liver fat amount.

In conclusion, telomere length tended to be shorter in NALFD patients, but the difference was not statistically significant. However, our findings confirmed the significant association between fibrosis severity and telomere length in NAFLD patients. Although this study had some limitations in clarifying all the associations between telomere and NAFLD, the relationship between fibrosis and telomere length was robustly confirmed. Therefore, sequel studies would be necessary to investigate the pathogenic mechanisms underlying the association between liver fibrogenesis and telomere shortening.

## Supplementary Information

Supplementary Information 1.

## Abbreviations

NAFLD: Non-alcoholic fatty liver disease
NAFL: Non-alcoholic fatty liver
NASH: Non-alcoholic steatohepatitis
HCC: Hepatocellular carcinoma
DM: Diabetes mellitus
BMI: Body mass index
HOMA-IR: Homeostasis model assessment of insulin resistance
MR-PDFF: Magnetic resonance imaging proton density fat fraction
PCNA: Proliferating cell nuclear antigen
ALT: Alanine aminotransferase
AST: Aspartate aminotransferase
γGT: Gamma-glutamyl transferase
ALP: Alkaline phosphatase
HDL: High-density lipoprotein
