# Supplementary material for: Association between telomere length and hepatic fibrosis in non-alcoholic fatty liver disease
Source: Sci Rep. 2021 Sep 9;11:18004. doi: 10.1038/s41598-021-97385-2 (PMC8429461; doi:10.1038/s41598-021-97385-2)
Supplement: Supplementary file 1 — Supplementary Information 1. [file 41598_2021_97385_MOESM1_ESM.docx]

**Supplementary Table 1. Histological characteristics of study subjects according to the age-adjusted mean telomere length**

|  | Total  (n=83) | <−0.013 Telomere  (n = 43) | | ≥−0.013 Telomere  (n = 40) | *P* value |
| --- | --- | --- | --- | --- | --- |
| Fibrosis stage, n (%) |  |  | |  | 0.043 |
| 0 | 23 (27.7) | | 6 (14.0) | 17 (42.5) |  |
| 1 | 32 (38.6) | | 18 (41.9) | 14 (35) |  |
| 2 | 20 (24.0) | | 13 (30.2) | 7 (17.5) |  |
| 3 | 3 (3.61) | | 2 (4.7) | 1 (2.5) |  |
| 4 | 5 (6.02) | | 3 (7.0) | 2 (5) |  |
| Steatosis grade, n (%) |  | |  |  | 0.079 |
| 0 | 23 (27.7) | | 9 (20.9) | 14 (35) |  |
| 1 | 10 (12.0) | | 4 (9.3) | 6 (15) |  |
| 2 | 15 (18.1) | | 12 (27.9) | 3 (7.5) |  |
| 3 | 35 (42.1) | | 18 (41.9) | 17 (42.5) |  |
| Lobular inflammation, n (%) |  | |  |  | 0.716 |
| 0 | 21 (25.3) | | 9 (20.9) | 12 (30) |  |
| 1 | 39 (47.0) | | 21 (52.5) | 18 (45) |  |
| 2 | 23 (27.7) | | 13 (30.2) | 10 (25) |  |
| 3 | 0 (0) | | 0 (0) | 0 (0) |  |
| Ballooning, n (%) |  | |  |  | 0.172 |
| 0 | 31 (37.3) | | 12 (27.9) | 19 (47.5) |  |
| 1 | 47 (56.6) | | 28 (65.1) | 19 (47.5) |  |
| 2 | 5 (6.02) | | 3 (7.0) | 2 (5) |  |

Mean telomere length = −0.013

**Supplemenraty Figure 1.**


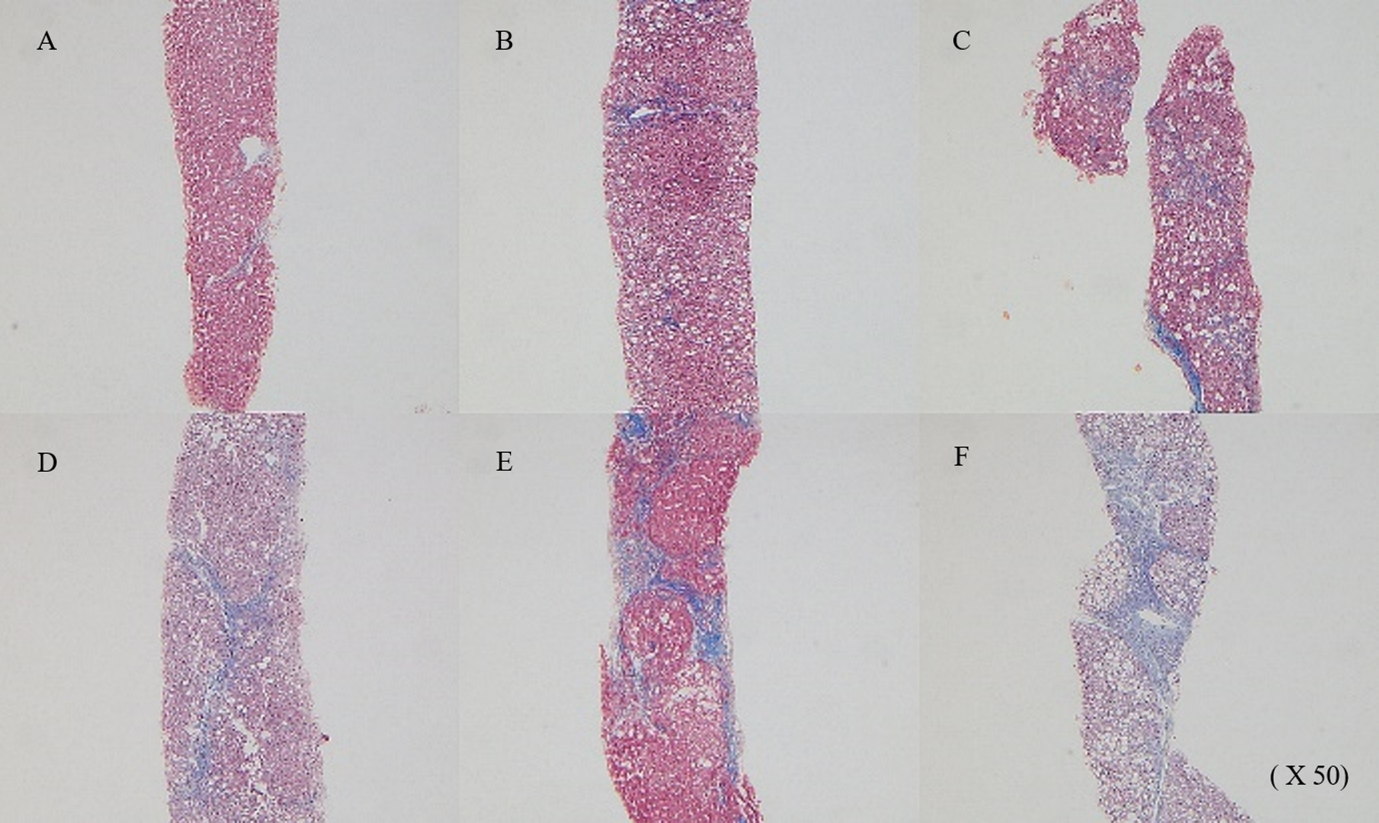


Supplementary Figure 1. Fibrosis stages in NAFL and NASH patients. (A) F0, (B) F1a, (C) F2, (D) F3, (E) F4A, (F) F4B (Original magnification, x50)

NAFL, non-alcoholic fatty liver; NASH, non-alcoholic steatohepatitis.

**Supplemenraty Figure 2.**


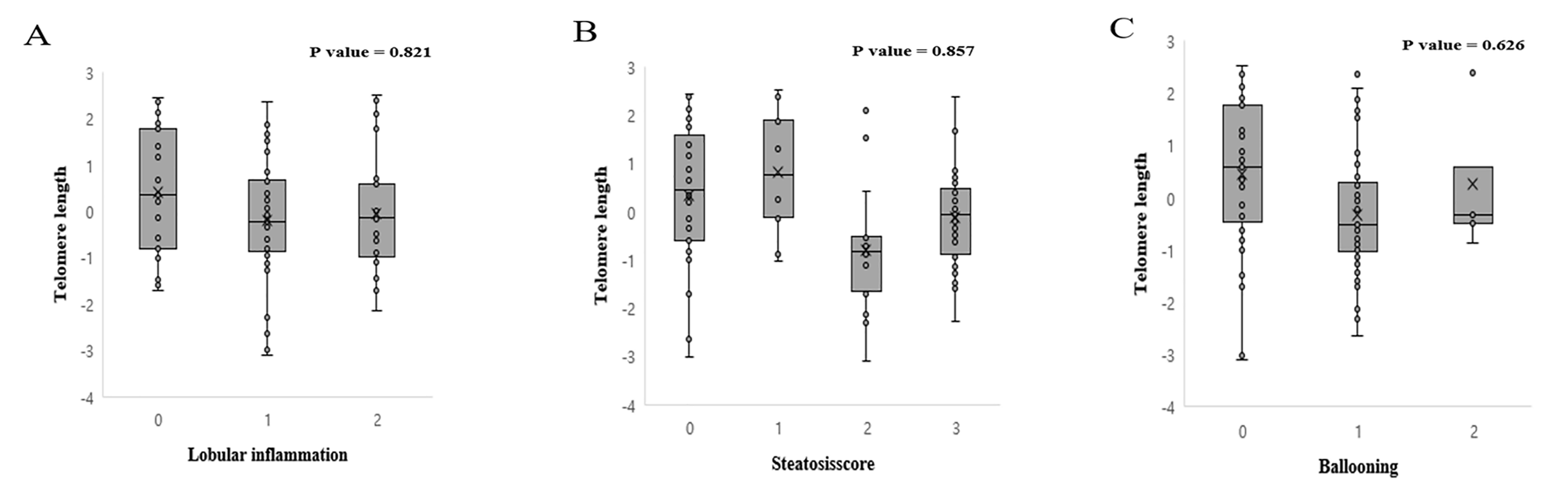


Supplementary Figure 2. Correlation between telomere length and other pathologic parameters in NAFLD patients. (A) Lobar inflammation, (B) Steatosis score, and (C) Ballooning.

NAFLD, non-alcoholic fatty liver disease
